# Echocardiographic parameters indicating left atrial reverse remodeling after catheter ablation for atrial fibrillation

**DOI:** 10.3389/fcvm.2023.1270422

**Published:** 2023-12-18

**Authors:** Eleonora Angelini, Jan-Thorben Sieweke, Dominik Berliner, Saskia Biber, Stephan Hohmann, Maximiliane Oldhafer, Sven Schallhorn, David Duncker, Christian Veltmann, Johann Bauersachs, Udo Bavendiek

**Affiliations:** ^1^ Department of Cardiology and Angiology, Hannover Medical School, Hannover, Germany; ^2^ Center for Electrophysiology, Klinikum Links der Weser, Bremen, Germany

**Keywords:** atrial fibrillation, 2D speckle tracking, atrial remodeling, atrial conduction time, pulmonary vein isolation, catheter ablation

## Abstract

**Background:**

The echocardiographic parameters total atrial conduction time (PA-TDI duration), left atrial (LA) volume index (LAVI), and LA strain reflect adverse atrial remodeling and predict atrial fibrillation (AF).

**Objectives:**

The aim of this study was to investigate echocardiographic parameters indicating reverse LA remodeling and potential associations with AF recurrence after pulmonary vein isolation (PVI).

**Methods:**

This prospective observational study consecutively enrolled patients scheduled for PVI for symptomatic AF. Electrocardiogram (ECG) test and transthoracic echocardiography were performed the day before and after PVI and again 3 months later. AF recurrence was determined by Holter ECG at 3 months, and telephone follow-up at 12 months, after PVI. The parameters of LA remodeling [PA-TDI, LAVI, and LA strain analysis: reservoir strain (LASr), conduit strain (LAScd), contraction strain (LASct)] were determined by transthoracic echocardiography.

**Results:**

A total of 48 patients were included in the study (mean age: 61.4 ± 12.2 years). PA-TDI significantly decreased the day after PVI compared with the baseline (septal PA-TDI 103 ± 13 vs. 82 ± 14.9 ms, *p* ≤ 0.001; lateral PA-TDI 122.4 ± 14.8 vs. 106.9 ± 14.4 ms, *p* ≤ 0.001) and at the 3-month follow-up (septal PA-TDI: 77.8 ± 14.5, *p* ≤ 0.001; lateral PA-TDI 105.2 ± 16.1, *p* ≤ 0.001). LAVI showed a significant reduction at the 3-month follow-up compared with the baseline (47.7 ± 14.4 vs. 40.5 ± 9.7, *p* < 0.05). LASr, LAScd, and LASct did not change after PVI compared with the baseline. AF recurred in 10 patients after PVI (21%). Septal PA-TDI, septal a', and LAVI/a' determined the day after PVI were associated with AF recurrence.

**Conclusion:**

Changes in echocardiographic parameters of LA remodeling and function indicate that functional electromechanical recovery preceded morphological reverse remodeling of the left atrium after PVI. Furthermore, these changes in echocardiographic parameters indicating LA reverse remodeling after PVI may identify patients at high risk of AF recurrence.

## Introduction

Catheter ablation has become a standard therapy for patients with symptomatic non-permanent atrial fibrillation (AF) (1). Catheter ablation of AF by pulmonary vein isolation (PVI) has rapidly developed in recent years after the first description of the role of pulmonary veins in triggering AF (2, 3). However, despite technical advances, successful maintenance of sinus rhythm (SR) in the first year after PVI is approximately 70% in paroxysmal AF and 50% in persistent AF (4–6).

AF causes electrical and structural left atrial (LA) remodeling, which contributes to the susceptibility to AF perpetuation and its progression (7, 8). Echocardiography is the most widely used modality to evaluate LA dimension and function before scheduling AF ablation. Echocardiographic parameters of LA remodeling comprise total atrial conduction time (PA-TDI interval), the ratio of LA volume indexed to tissue Doppler a' (LAVI/a'), and left atrial strain (9). PA-TDI indicates left atrial electromechanical coupling (10). PA-TDI and LAVI/a' have been shown to predict incident AF in different patient populations in SR (8–12). A reduced LA strain is associated with an increased risk of AF occurrence and recurrence of cerebral infarction (13).

The mechanisms explaining a reduction in LA dimensions after PVI have been described in basic science studies, showing reverse atrial remodeling following restoration of sinus rhythm as occurs following PVI (14). However, there are no data available about a potential change and time-course of the echocardiographic parameters PA-TDI, LAVI/a', and LA strain after PVI. Therefore, the present study investigated whether PVI affects echocardiographic parameters of LA remodeling and function indicating reverse LA remodeling after restoration of SR.

## Methods

### Study design and participants

**Figure 1:**
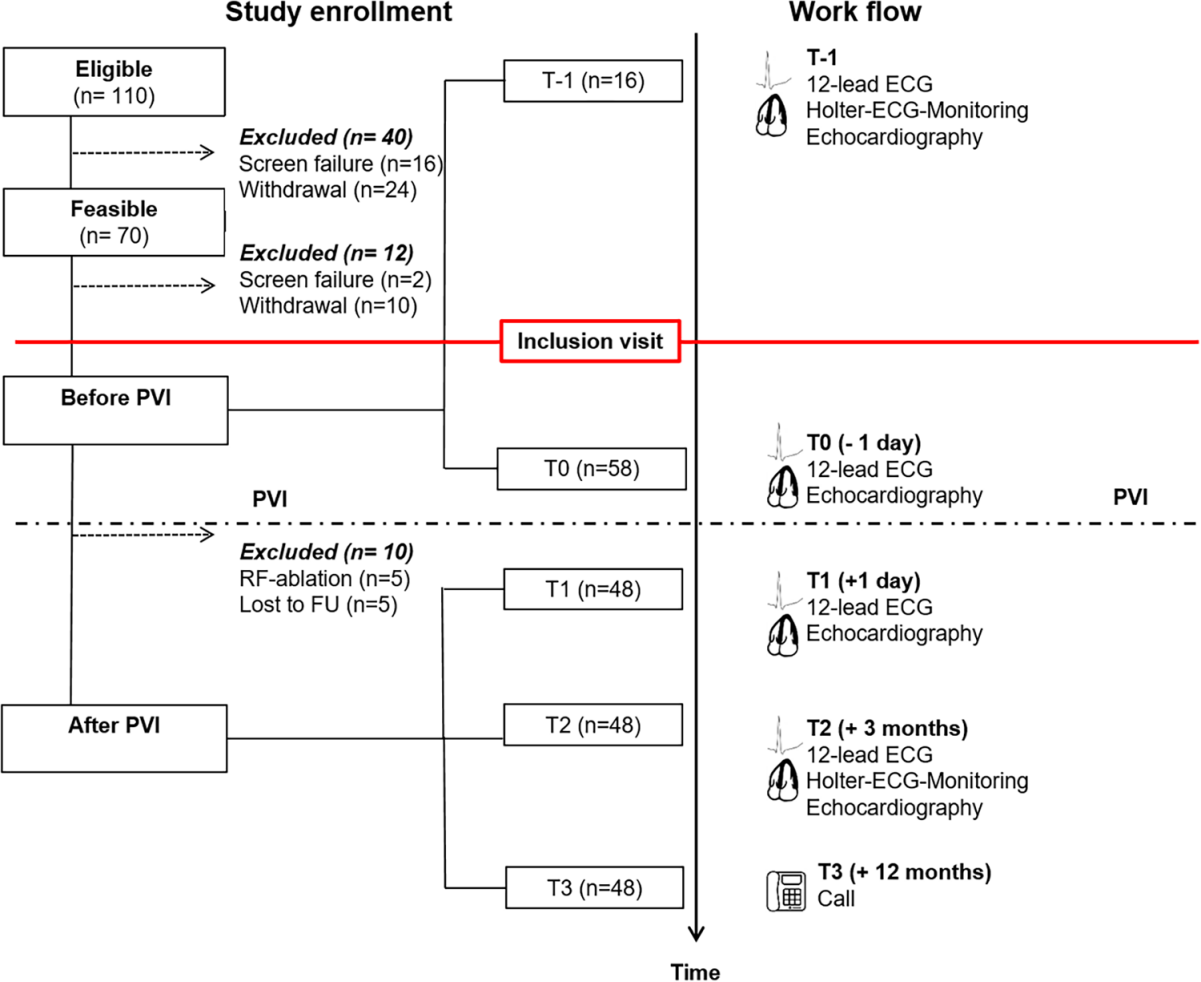
Study enrollment and work flow. FU, follow-up; RF, radio-frequency.

The present study is a prospective, longitudinal, single-center study, which was approved by the local ethics committee of the Hannover Medical School (# 7910_BO_S_2018). The study complies with the Declaration of Helsinki. All participants gave their written informed consent. We consecutively screened patients for eligibility, selecting those who had undergone PVI due to symptomatic AF in the Department of Cardiology and Angiology between June 2018 and July 2019. Of importance, patients were only included in this study (T0) if they presented in SR during the echocardiographic examination because parameters for LA remodeling cannot be determined during AF. The exclusion criteria were defined as follows: age less than 18 years, severe mitral valve stenosis or regurgitation, history of aortic or mitral valve replacement, AF during echocardiography, hyperthyroidism, previously diagnosed channelopathies, alcohol abuse, contraindications against PVI, active devices (left ventricular assist device, pacemaker, implantable cardioverter-defibrillator, cardiac resynchronization therapy), patients lost to follow-up, radio-frequency (RF) catheter ablation, and patients who could not provide informed consent. In particular, RF ablation was an exclusion criterion because all included patients underwent AF ablation for the first time, and to strengthen the primary endpoint in a homogeneous patient cohort. Inclusion of the participants and workflow are presented in Figure 1.

### Echocardiography

Standard transthoracic echocardiography was performed according to the American Society of Echocardiography guidelines at inclusion visit (T0) and in the follow-up (T1, T2) as described in Figure 1 (15). In addition, specific echocardiographic parameters reflecting LA function and remodeling were determined (7, 15). Left atrial volume was determined by biplane area length method in apical four- and two-chamber views at the ventricular end-systole from the apical approach in brief breathhold (15). Subsequently, left atrial volume was indexed (LAVI) to body surface area (BSA). Using tissue Doppler imaging, septal and lateral late diastolic peak tissue Doppler velocity (a') was determined. The average of the septal and lateral a' was used to assess LAVI/a'. In tissue Doppler imaging, the interval between the onset of P wave in lead II of the ECG and the peak A'-wave of the septal or lateral mitral valve (MV) annulus was defined as septal or lateral PA-TDI. Two-dimensional (2D) speckle tracking analyses of the left atrium were conducted offline with customized computer software (TOMTEC Autostrain for LA; TOMTEC Imaging Systems GmbH, Unterschleissheim, Germany) using zoom mode images of the LA in four- and two-chamber views to obtain LA reservoir, conduit, and contraction strains (LASr, LAScd, LASct). Investigators analyzing the echo images were not involved in PVI and were unaware of patients' ECG analyses. To evaluate the accuracy of the echocardiographic parameters and to exclude a change of the echocardiographic parameters over time, we compared echocardiographic examinations of the inclusion visit (T0) with echocardiographic examinations conducted before study inclusion in the outpatient clinic (T1) if available in a subpopulation of patients.

### ECG and Holter ECG monitoring

All participants received 12-lead ECG at inclusion and follow-up (T1, T2). In addition, 24 h Holter was performed at 3 months after PVI (T2). All ECG recordings were analyzed during clinical routine by blinded professionals applying current guidelines on AF (1).

### Pulmonary vein isolation and definitions

Pre-procedure transesophageal echocardiography was performed to assess the left atrium, left atrium appendage, and pulmonary vein anatomy. Cryoballoon ablation was performed using a 28-mm cryoballoon (Artic Front Advance; Medtronic) using standard protocols to achieve persistent bidirectional conduction block in all pulmonary veins. During the procedure, esophageal temperature was monitored (CIRCA S-Cath, Circa Scientific Inc.). After circumferential antral vein isolation, a 30-min waiting period was conducted to assess spontaneous conduction recovery. Pericardial effusion was ruled out after PVI by echocardiography.

### Follow-up

Follow-ups were performed after PVI during in-hospital stay (T1), in the outpatient clinic after 3 months (T2), and by telephone call 12 months (T3) after catheter ablation. Transthoracic echocardiography and 12-lead ECG were scheduled for T1 and T2. In addition, Holter ECG was performed at the follow-up 3 months after PVI (T2). The follow-up visit after 12 months (T3) was performed by telephone call. AF recurrence was defined by AF detection in Holter ECG at the 3-month follow-up and telephone follow-up 12 months after PVI (Figure 1).

### Study outcomes

The primary endpoint of the analysis was the change in the total atrial conduction time (PA-TDI interval) assessed by transthoracic echocardiography, indicating LA remodeling after PVI. Key secondary endpoints included change in the ratio of the indexed left atrial volume and mitral annulus velocity during atrial contraction a' (LAVI/a'), change in LA dimension, and changes in 2D speckle tracking parameters of the left atrium (LA strain), which also indicate LA remodeling.

### Statistical analysis

Statistical analysis and graphical presentation were performed using SPSS Statistics 27 (IBM SPSS Statistics 27) and GraphPad Prism 7.04 (GraphPad Software, San Diego, CA, USA). Categorical variables are presented as *n* (%). Normally distributed continuous variables are given as mean ± standard deviation (SD), or median and interquartile ranges (IQR) for non-normally distributed variables. Normality and variance homogeneity were checked by the Shapiro–Wilk test. Comparison between the groups was performed using the Student's *t*-test for Gaussian distributed data and the Mann–Whitney *U* test for non-normally distributed data. ANOVA was performed followed by Bonferroni test or Dunn's test for multiple comparisons, respectively. The categorical variables were evaluated by the chi-square test. A two-sided *p*-value of <0.05 was considered statistically significant. In a sample size analysis before the start of the study, based on the publication by Fukushima et al. (16), a number of 44 patients was calculated under the condition of a power of 80% in order to achieve a two-sided significance level of 5% (*p* = 0.05). The discriminative ability of septal PA-TDI at T1 was assessed by the area under the receiver operating characteristic (ROC) curve. The prediction values were defined as the cut-off point having the highest Youden index (Yi = sensitivity + specificity − 1). The sensitivity, specificity, and accuracy for determining cut-offs were calculated.

## Results

### Patient enrollment and characteristics

Between June 2018 and July 2019, a total of 58 of the 110 eligible patients were included in the study after applying the inclusion and exclusion criteria. Of these patients, 48 had a transthoracic echocardiography and an ECG taken one day after PVI. At the second visit three months after PVI, all 48 patients participated. Furthermore, all 48 patients were available at a telephone visit after 12 months. The study flow is presented in Figure 1.

Baseline characteristics of the study patients are summarized in Table 1. The patients with symptomatic paroxysmal AF (EHRA 2a-3) had a mean age of 61.4 ± 12.2 years, and 66% were male. Hypertension was apparent in 33 patients (69%). All study participants underwent first-time PVI using a cryoballoon. Primary successful isolation of all pulmonary veins was performed in all patients. No complications occurred. The medical treatment is presented in Supplementary Table S3.

**Table 1:** Baseline characteristics at inclusion.

Parameter	In-hospital inclusion visit
	T0 *n* = 48
Age (years)	61.4 ± 12.2
Sex: male	31 (66%)
Height (cm)	176.8 ± 10.7
Weight (kg)	86 ± 17.6
Pre-existing conditions	
Hypertension	33 (69%)
Diabetes	5 (10%)
Stroke	2 (4%)
CAD^a^	11 (23%)
Nicotine	13 (27%)
PAD^b^	1 (2%)
EHRA	III (II–III)
CHADS_2_	
0	13 (27%)
1	15 (31%)
2	18 (38%)
3	2 (4%)
4	0
5	0
CHA_2_DS_2_-VASc	
0	10 (21%)
1	7 (16%)
2	11 (23%)
3	9 (19%)
4	10 (21%)
5	1 (2%)
NT-proBNP (mmol/L)	122 (68–282)
PVI^c^ characteristics	
Number of applications	7 (5–9)
Duration per application (s)	180 (180–198)

^a^ Coronary artery disease.

^b^ Peripheral arterial disease.

^c^ Pulmonary vein isolation.

Variables are expressed as mean ± SD, median (IQR), or *n* (% of total number). CHA2DS_2_-VASc score and CHADS_2_ score were determined on medical history at inclusion to the study.

### Echocardiographic parameters of LA remodeling and fibrosis

**Figure 2:**
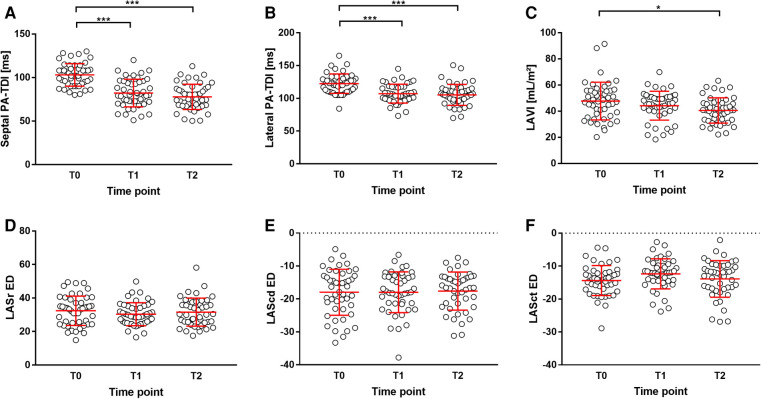
Parameters of LA remodeling during study. (**A**) septal PA-TDI, (**B**) lateral PA-TDI, (**C**) LAVI, (**D**) LASr ED, (**E**) LAScd ED, and (**F**) LASct ED. *p < 0.05; ***p < 0.001. LAScd ED, left atrial conduit strain at end-diastole; LASct ED, left atrial contractile strain at end-diastole; LASr ED, left atrial reservoir strain at end-diastole.

The parameters of echocardiography are summarized in Table 2. Patients had a normal left ventricular ejection fraction with a mean of 60.9 ± 4.9%. After PVI, septal PA-TDI and lateral PA-TDI were significantly reduced in the follow-up visits T1 and T2 compared with T0 (Figures 2A,B). By contrast, LAVI did not change at in-hospital visit T1, although it was significantly reduced compared with the baseline at follow-up visit T2 at 3 months after PVI (Figure 2C). The LA strain parameter did not significantly change over the course of the entire follow-up period after PVI (Figures 2D–F). Of note, the internal control group showed that echocardiographic parameters of LA remodeling and function were not significantly altered over time without PVI intervention (T0 vs. T-1) as presented in Supplementary Table S1.

**Table 2:** Characteristics of transthoracic echocardiography.

Parameter	In-hospital inclusion visit	In-hospital	Outpatient clinic
	T0 *n* = 58	T1 *n* = 51	T2 *n* = 56
Interval from TTE to PVI (days)	1 ± 0	2 (2–3)	94 (92–103)
LVEF (%)	60.9 ± 4.9	61 ± 4.3	60.1 ± 4.4
Diameter LV^a^ (cm)	4.9 ± 0.4	4.8 ± 0.5	4.8 ± 0.6
Diameter RV^b^ (cm)	3.7 ± 0.4	3.6 ± 0.4	3.6 ± 0.5
E' septal (cm/s)	6.7 ± 1.6	6.6 ± 1.7	6.6 ± 1.6
E' lateral (cm/s)	9.1 ± 2.6	8.6 ± 3.1	8.6 ± 2.6
E/E' septal	11.4 (9.3–13.6)	12.1 (9.4–15.8)	11 (8.9–14.2)
E/E' lateral	8.2 (6.5–11.4)	10 (7.1–12.1)	8.6 (6.7–11.1)
a' septal (cm/s)	8.5 ± 1.8	8.4 ± 2.1^‡^	8.3 ± 2
a' lateral (cm/s)	8.7 ± 2.7	8.7 ± 2.9	8.7 ± 2.5
MV^c^ E/A	1.1 (0.8–1.5)	1.1 (1–1.6)	1.2 (1–1.5)
PA-TDI septal (ms)	103 ± 13	82 ± 14.9**^,^^§^	77.8 ± 14.5**
PA-TDI lateral (ms)	122.4 ± 14.8	106.9 ± 14.4^**^	105.2 ± 16.1**
LAVI^d^ (ml/m²)	47.7 ± 14.4	44.2 ± 11.1	40.5 ± 9.7*
LAVI/a'	5.5 (4.4–6.6)	5.5 (4.1–6.8)^‡^	5.1 (3.5–6.7)
LA diameter	4.3 ± 0.5	4.2 ± 0.5	4.2 ± 0.5
2D speckle tracking strain analysis of LA^e^			
LASr	32.3 ± 8.7	30.4 ± 6.8	31.5 ± 8.4
LAScd	−18 ± 7	−18 ± 6.2	−17.6 ± 5.8
LASct	−14.3 ± 4.6	−12 ± 4.6	−13.9 ± 5.4

^a^ Left ventricle.

^b^ Right ventricle.

^c^ Mitral valve.

^d^ Left atrial volume index.

^e^ Left atrium.

* *p* < 0.05 vs. T0.

** *p* < 0.001 vs. T0.

^‡^ *p* < 0.01 vs. AF recurrence after pulmonary vein isolation.

^§^ *p* < 0.01 vs. AF recurrence after pulmonary vein isolation.

### Association of echocardiographic parameters in AF recurrence

Overall, 10 patients had a recurrence of AF after PVI (21%) within the 12-month follow-up. After 3 months (T2), nine patients showed AF in a 12-lead ECG or in a Holter ECG. At the telephone follow-up visit (T3) 12 months after ablation, one patient reported symptomatic documented recurrence of AF.

**Figure 3:**
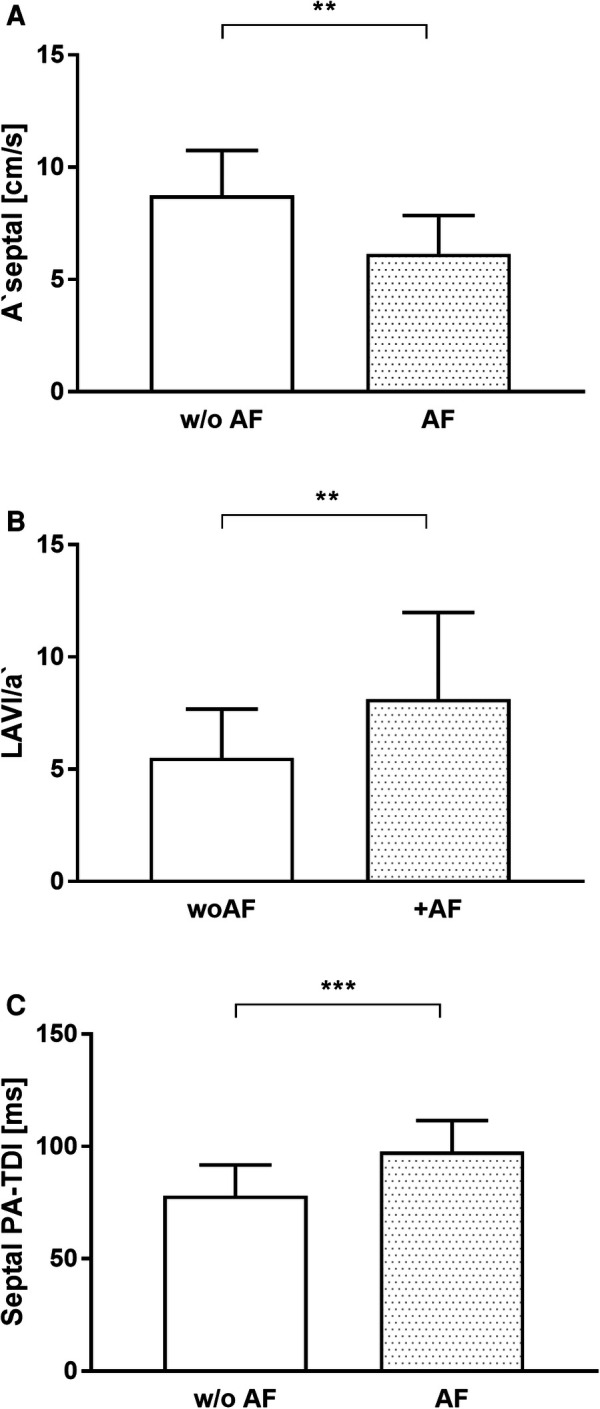
Echocardiographic parameters of LA remodeling and function associated with the recurrence of AF. (**A**) A ‘septal, (**B**) LAVI/a’, (**C**) Septal PA-TDI. AF, atrial fibrillation; w/o AF, without atrial fibrillation. **p < 0.01; ***p < 0.001.

There were no differences in echocardiographic baseline characteristics between patients with and without recurrence of AF (Table 2). Septal PA-TDI, LAVI/a', and A' septal—echocardiographic parameters of LA remodeling, dimension, and function, respectively—differed significantly in patients with AF recurrence after PVI (T1) (Figure 3). The ROC analysis and subsequent analysis of discriminators identified a septal PA-TDI at T1 of 87 ms as optimal cut-off for the prediction of AF recurrence with a specificity of 74% and sensitivity of 80% (Supplementary Figure S1).

## Discussion

In the present study, for the first time the change of echocardiographic parameters indicating reverse LA remodeling after PVI over time was described. Immediately after PVI, there was a change in electromechanical coupling as indicated by PA-TDI. Delayed reverse remodeling led to a significant reduction in LA dimensions illustrated by LAVI. This study indicated that functional electromechanical recovery precedes morphological reverse remodeling of the LA after PVI. However, there was no statistically significant improvement in LA function assessed by LAVI/a' and LA strain during the 3 months follow-up period.

LA remodeling is crucial for the development and persistence of AF and primarily consists of electrical, contractile and structural remodeling processes (17). Furthermore, to increase the success rate of PVI and to enable enhanced patient selection, it is important to understand LA remodeling in AF more precisely and thereby achieve optimal treatment planning and risk stratification. Transthoracic echocardiography, a widely available non-invasive and accurate imaging approach, allows determination of parameters associated with structural processes in LA remodeling. Structural remodeling is associated with dilatation and progressive fibrosis of the left atrium, and the electrical remodeling process is manifested by a shortening of the action potential and atrial refractory period (18).

Of note, in the present study septal PA-TDI after PVI was longer in the AF recurrence group than in the group without AF recurrence. The significant reduction in PA-TDI duration occurred immediately after successful PVI and persisted 3 months later indicating early functional electromechanical recovery. den Uijl et al. (19) described that PA-TDI duration before PVI is an independent predictor of AF recurrence after PVI. Due to the lower sample size calculated for the primary hypothesis in the present study compared with den Uijl et al., the number of patients may not be sufficient to calculate predictions for AF recurrence. However, the septal PA-TDI, collected after PVI at time T1, showed a high degree of discriminatory ability between patients with recurrence of AF and patients without recurrence [area under the curve (AUC): 0.853]. The ROC analysis followed by determination of Youden’s index calculated a separation value of 87 ms (sensitivity 80%, specificity 74%) for septal PA-TDI after PVI (T1) associated with recurrence of AF (Supplementary Figure S1). Although our current study pursued a different primary objective, the power analysis based on our results generate the hypothesis that septal PA-TDI after PVI could be used as a parameter for risk stratification of AF recurrence.

LA enlargement is known to be characteristic in patients with AF as well as a predictor for AF occurrence (20, 21). Current guidelines recommend the measurement of LA volume indexed to BSA when assessing the LA size, since indexing accounts for the gender differences in LA size and hence provides comparable values (13). The ratio of LAVI to LAVI/a' has been demonstrated to allow patients with arterial hypertension and AF to be distinguished from those without AF more precisely than LA size alone (8). In addition, LAVI/a’ was demonstrated to be an independent predictor of AF in patients with cryptogenic cerebral infarction (9). In our analysis, LAVI was significantly reduced 3 months after PVI. Furthermore, LAVI/a' was significantly greater in patients with AF recurrence after PVI compared with patients without AF recurrence. LA size, assessed by LAV and/or LAVI, is an independent predictor of maintenance of SR after catheter ablation of AF (17, 21). In our analysis, PVI was followed by a decrease in LA size, which correlated with persistence of SR.

LA strain correlates with the progress of LA fibrosis and hence impaired function in AF (7, 22). Alterations in strain rate analysis are predictive for successful ablation of AF during follow-up (23). This evidence is supported by Tops et al. describing a significant increase in LA strain, measured by TDI suggesting reverse remodeling 12 months after PVI (24). In our speckle tracking analysis, LA strain parameters did not change significantly after PVI within the 3-month follow-up presumably because reversal of LA fibrosis reflected by improvement of LA strain parameters takes longer as indicated by Tops et al. (24).

In our study, the PVI success rate of 79% is within the range known from the literature (62%–86% during a follow-up of 6–12 months) (25). PVI was considered successful if patients’ symptoms improved and a Holter ECG and 12-lead ECG showed SR 3 months after PVI. In the present study, echocardiography did not demonstrate maladaptive consequences of PVI in patients who had AF recurrence. Our results corroborate the work of Verma et al. (26), who also excluded any impairment of LA function after PVI.

Previous studies (27, 28) reported that LA remodeling is partially reversible: in the context of electrophysiological studies during PVI, the electrical changes might completely regress after termination of atrial tachycardia, whereas structural remodeling reverts slower or not at all depending on AF burden. Our findings support this assumption and consolidate the value of echocardiographic assessment of LA remodeling, especially with regard to septal PA-TDI. In addition, parameters used to assess LA remodeling and LA function, particularly septal PA-TDI and LAVI/a', showed little variance over time. This finding reinforces the importance of using these parameters to optimize the risk stratification of patients with AF.

The following aspects may limit our findings: (1) The sample size was tailored for the primary endpoint (change in the total atrial conduction time) and required a low number of patients enrolled. The analysis was not aimed to accurately investigate the change of other echocardiographic parameters, which also reflect adverse atrial remodeling. (2) We adjudicated PVI to be successful if patients’ symptoms improved and a Holter ECG and 12-lead ECG showed SR 3 months after PVI. However, we did not assess AF burden after ablation. (3) After pulmonary vein isolation, there was significantly more frequent use of non-vitamin K antagonists, especially apixaban (Supplementary Table S3). In animal models, a pleiotropic effect of another anti-Xa antagonist, edoxaban, was recognized with less progression of fibrotic progression of the atrium during tachypacing (29). However, it is puzzling whether this is a class effect that also occurs in humans and influences reverse remodeling. (4) In the follow-up at the time point of echocardiographic examination, an electrophysiological study to assess atrial reconduction would have been most accurate. However, like other authors, we are convinced that assessment of recurrent atrial fibrillation by long-term ECG is sufficient for this question. This is especially justifiable under consideration of ethics and patient safety. (5) Furthermore, the underlying mechanisms causal for the reduction of PA-TDI duration immediately after PVI remains open. Future studies should address this issue.

## Clinical perspectives

To increase the success rate of PVI and to enable enhanced patient selection, it is important to understand LA remodeling in AF more precisely and thereby achieve optimal treatment planning and risk stratification. Transthoracic echocardiography, a widely available, non-invasive, and accurate imaging approach, allows the determination of parameters associated with structural processes in LA remodeling. Based on our data, future studies should investigate potential associations of echocardiographic parameters indicating LA reverse remodeling with AF recurrence after PVI.

## Conclusions

Changes of echocardiographic parameters after PVI indicate that functional electromechanical recovery precedes morphological reverse remodeling of the left atrium. Our data provide the basis for further studies that investigate potential associations of echocardiographic parameters indicating LA reverse remodeling with AF recurrence after PVI to optimize the selection of PVI candidates using echocardiographic parameters and to identify patients at high risk of AF recurrence after PVI.

## Data Availability

The original contributions presented in the study are included in the article/**Supplementary Material**, further inquiries can be directed to the corresponding author.

## Author’s note

This manuscript is part of the doctoral thesis of Eleonora Angelini supervised by Jan-Thorben Sieweke.
