# Supplementary material for: Echocardiographic parameters indicating left atrial reverse remodeling after catheter ablation for atrial fibrillation
Source: Front Cardiovasc Med. 2023 Dec 18;10:1270422. doi: 10.3389/fcvm.2023.1270422 (PMC10757954; doi:10.3389/fcvm.2023.1270422)
Supplement: Supplementary file 1 [file Datasheet1.pdf]

Supplemental Table 1: Internal Validation- Echocardiography

**Parameter**	**Out-patient clinic**<br><br>T-1<br>n=16	**In-hospital**<br>Inclusion visit<br><br>T0<br>n=16	***p***
LVEF [%]	59.4±5.3	59.4±3.8	ns
Diameter LV [cm]	4.9±0.5	5.0±0.4	ns
Diameter RV [cm]	3.5±0.3	3.6±0.4	ns
A`septal [cm/s]	8.3±1.9	8.2±1.7	ns
A`lateral [cm/s]	8.9±3.1	8.1±3.0	ns
PA-TDI septal [ms]	110.5±16.9	111.5±17.1	ns
PA-TDI lateral [ms]	130±13.9	130.3±11.7	ns
LAVI [ml/m²]	42.8±12.3	42.8±12.3	ns
LAVI/a`	5.3 [4.3-6.8]	5.6 [4.4-6.3]	ns
Longitudinal strain analysis of LA:			
LASr	31.6±9.5	30.7±8.4	ns
LAScd	-18.1±7.5	-18.0±7.0	ns
LASct	-13.6±4.7	-13.3±4.1	ns
LA diameter	4.3±0.5	4.3±0.5	ns

AF- Atrial fibrillation, LA- left atrium, LAScd- left atrial conduit strain, LASct left atrial contractile strain, LASr- left atrial reservoir strain, LAVI- left atrial volume index, LV- left ventricle, LVEF- left ventricular ejection fraction, MV- mitral valve, RV- right ventricle. Variables are expressed as mean±SD or median [IQR].

Supplemental Table 2: Baseline characteristics of patients with and without recurrence of AF

**Parameter**	**w/o recurrence of AF** n=38	**recurrence of AF** n=10	***p***
Age [years]	61.8±11.2	58.1±15.4	ns
Sex: Male	25 (66%)	6 (60%)	ns
Height [cm]	176.7±11.2	177±12.4	ns
Weight [kg]	85±16.4	90.7±20.1	ns
Pre-existing conditions			
Hypertension	27 (71%)	6 (60%)	ns
Diabetes	4 (11%)	1 (10%)	ns
Stroke	0	2 (20%)	ns
CAD[a]	8 (21%)	3 (30%)	ns
Nicotine	11 (29%)	2 (20%)	ns
PAD[b]	1 (3%)	0	ns
In-hospital stay [days]	3 [3-3]	4 [3-4]	ns
EHRA	III [II-III]	III [II-III]	ns
CHADS$_2$			ns
0	10 (26%)	3 (30%)	
1	13 (34%)	2 (20%)	
2	14 (37%)	4 (40%)	
3	1 (3%)	1 (10%)	
CHA$_2$DS$_2$VASc			ns ns
1	17 (45%)	4 (40%)	
2	6 (16%)	1 (10%)	
3	8 (21%)	1 (10%)	
4	7 (18%)	3 (30%)	
5	0	1 (10%)	
NT-proBNP [mmol/l]	122 [57-271]	168 [45-344]	ns
PVI[d] characteristics			
Number of applications	6 [5-9]	7 [6-9]	ns
Duration per application [sec]	180 [180-210]	210 [180-240]	ns

[a] Coronary artery disease, [b] Peripheral arterial disease, [c] Atrial fibrillation, [d] Pulmonary vein isolation; Variables are expressed as mean±SD, median [IQR] or n (% of total number). CHA2DS2-VASc Score and CHADS2-Score was determined on medical history at inclusion to the study.

Supplemental Table 3: Medical treatment

**Parameter**	**T1**<br>n=48	**T2**<br>n=48	***p***
Vitamin K antagonists	4 (8%)	4 (8%)	ns
NOAC	35 (73%)	44 (92%)	<0.05
Statine	15 (31%)	19 (39%)	ns
ACE-inhibitors	16 (33%)	19 (39%)	ns
ARB	13 (27%)	14 (29%)	ns
ß-blockers	35 (73%)	33 (69%)	ns
MRA	4 (8%)	4 (8%)	ns
Diuretics (loop)	13 (27%)	13 (27%)	ns

ARB- angiotensin receptor-blocker, MRA- mineralocorticoid receptor antagonist, NOAC- non-vitamin K antagonists, Variables are expressed as n (% of total number).
