# Supplementary figures and images for: Echocardiographic parameters indicating left atrial reverse remodeling after catheter ablation for atrial fibrillation
Source: Front Cardiovasc Med. 2023 Dec 18;10:1270422. doi: 10.3389/fcvm.2023.1270422 (PMC10757954; doi:10.3389/fcvm.2023.1270422)

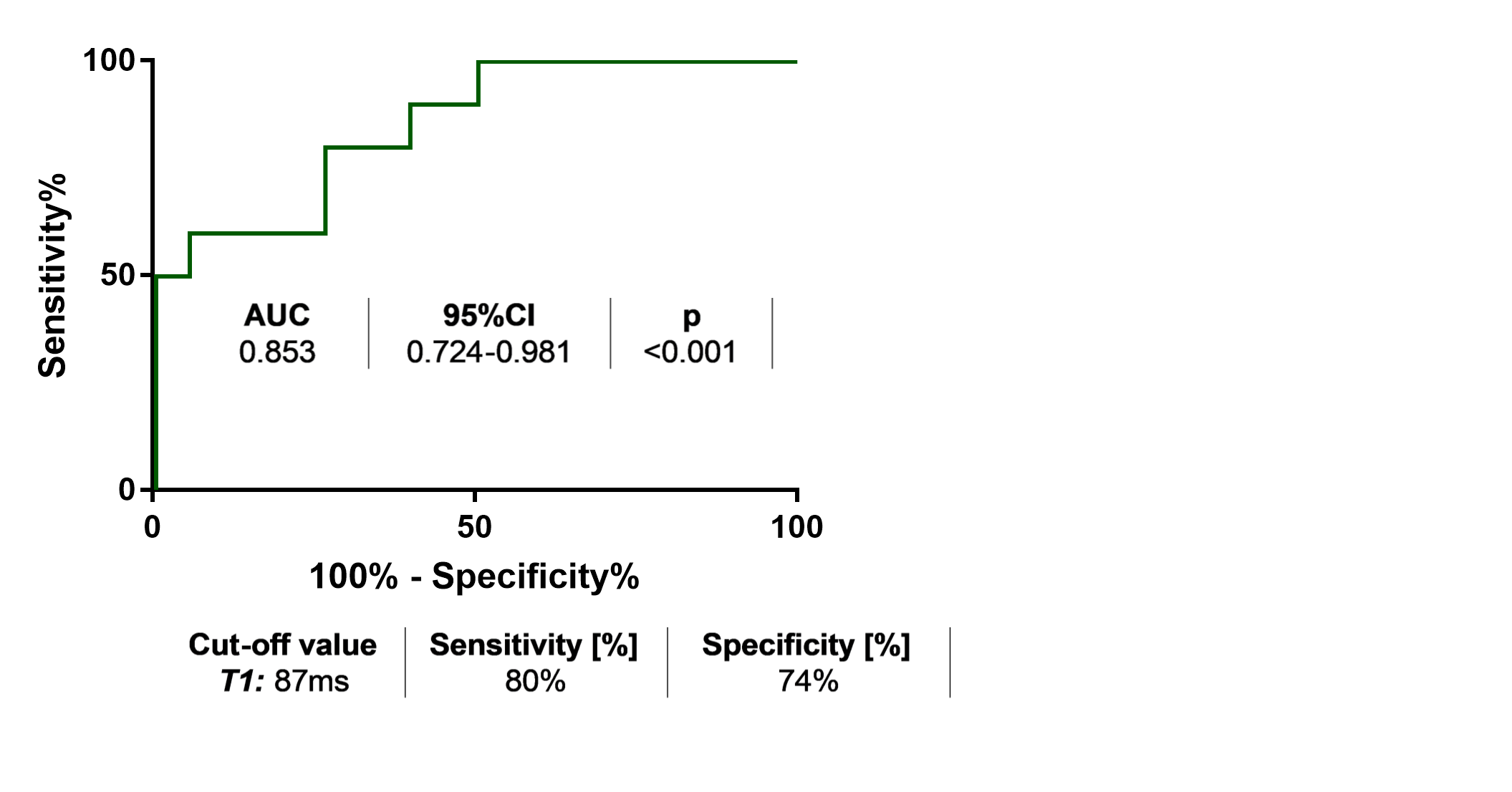

Supplement: Supplementary file 2 [file Image1.tif]
